# Clinicopathological Factors as Predictors for Establishment of Patient Derived Head and Neck Squamous Cell Carcinoma Organoids

**DOI:** 10.1007/s12105-024-01658-x

**Published:** 2024-06-28

**Authors:** W. W. B. de Kort, R. Millen, E. Driehuis, L. A. Devriese, R. J. J. van Es, S. M. Willems

**Affiliations:** 1 Department of Oral and Maxillofacial Surgery, University Medical Center Utrecht, 3584 CX Utrecht, The Netherlands; 2 Oncode Institute, Hubrecht Institute, Royal Netherlands Academy of Arts and Sciences (KNAW) and University Medical Center Utrecht, 3584 CT Utrecht, The Netherlands; 3 Department of Medical Oncology, University Medical Center Utrecht, Utrecht, The Netherlands; 4 Department of Head and Neck Surgical Oncology, Utrecht Cancer Center, University Medical Center Utrecht, 3584 CX Utrecht, The Netherlands; 5 Department of Pathology and Medical Biology, University Medical Center Groningen, 9713 GZ Groningen, The Netherlands

**Keywords:** Head and neck cancer, Patient derived organoids, Head and neck squamous cell carcinoma organoids, Clinicopathological parameters

## Abstract

**Introduction:**

Patient derived organoids (PDOs) are 3D in vitro models and have shown to better reflect patient and tumor heterogeneity than conventional 2D cell lines. To utilize PDOs in clinical settings and trials for biomarker discovery or drug response evaluation, it is valuable to determine the best way to optimize sample selection for maximum PDO establishment. In this study, we assess patient, tumor and tissue sampling factors and correlate them with successful PDO establishment in a well-documented cohort of patients with head and neck squamous cell carcinoma (HNSCC).

**Methods:**

Tumor and non-tumorous adjacent tissue samples were obtained from HNSCC patients during routine biopsy or resection procedures at the University Medical Center Utrecht. The tissue was subsequently processed to establish PDOs. The sample purity was determined as the presence of epithelial cells in the culture on the day of organoid isolation as visualized microscopically by the researcher. PDO establishment was recorded for all samples. Clinical data was obtained from the medical records and was correlated to PDO establishment and presence of epithelial cells.

**Results:**

Organoids could be established in 133/250 (53.2%) primary tumor site tissues. HNSCC organoid establishment tended to be more successful if patients were younger than the median age of 68 years (74/123 (60.2%) vs. 59/127 (46.5%), *p* = 0.03). For a subset of samples, the presence of epithelial cells in the organoid culture on the day of organoid isolation was recorded in 112/149 (75.2%) of these samples. When cultures were selected for presence of epithelial cells, organoid establishment increased to 76.8% (86/112 samples).

**Conclusion:**

This study found a trend between age and successful organoid outgrowth in patients with HNSCC younger than 68 years and emphasizes the value of efficient sampling regarding PDO establishment.

**Supplementary Information:**

The online version contains supplementary material available at 10.1007/s12105-024-01658-x.

## Introduction

The 5-year survival rates of head and neck squamous cell carcinoma (HNSCC) have only modestly improved over the past three decades from 55 to 66% [1]. Numerous predictive and prognostic biomarkers have been investigated to predict survival and guide treatment decisions [2–4]. However many of these biomarkers have been investigated using traditional 2D cell line models, which do not harbor the complex genetic and phenotypic heterogeneity that exists in these tumors in vivo. Therefore, there is a need to improve in vitro models to validate biomarkers that better reflect patient and tumor heterogeneity more accurately. Patient derived organoids (PDO) may fill this gap.

Organoids are microscopic 3D structures that can be grown from patient derived stem cells of healthy or tumor tissues [5]. Organoids were first established from intestinal epithelium, and replicated the morphology of the crypt-villus structures present in vivo, demonstrating the ability to recapitulate the native tissue pathophysiology in vitro [6–8]. For several tumor types, living biobanks of PDO’s have been established [9–14] and correlations between patient– and PDO drug response have been reported [6, 15–19].

Although PDOs have promising potential for personalized medicine, establishing PDOs can be laborious and costly. PDO establishment is more time-consuming compared to conventional 2D cell lines, and technically more difficult, requiring trained personnel [20, 21]. To utilize PDOs in clinical trials, it is important to know the success rates of establishing PDOs and if this correlates to clinical factors associated with the patient they are derived from. The reported pooled success rates for PDO establishment from multiple tissue and tumor types varies from 56.5 to 78.5% [16]. Herein, we assessed the correlation of patient tissue sampling and tumor-factors to PDO establishment in a previously published cohort of HNSCC patients [6, 15].

## Methods

### Patients and Clinical Data

This study analyzed organoids derived from a prospective cohort of patients with cancers of the head and neck area in the University Medical Center Utrecht (UMCU) as described in previously [6, 15]. The study protocol was approved by the Biobank Research Ethics Committee of the University Medical Center Utrecht (12-093 HUB-Cancer). All donors participating in this study signed informed-consent forms and could withdraw their consent at any time. Informed consent was obtained before tissue acquisition, patients were given a minimum of 24 hours to consider participation.

Patients were eligible for inclusion if (1) patients gave consent for the 12-093 HUB-cancer protocol, (2) patients had a type of HNSCC, (3) tissue acquisition was successful during biopsy for diagnostic histopathology or resection, (4) the laboratory of the Hubrecht institute tried to establish organoids for the sampled tissues.

### Tissue Acquisition

Primary tumor and/or lymph node metastatic tissue and tumor adjacent non-malignant tissue was obtained from HNSCC patients during either biopsy or resection procedures as part of their routine diagnostic or treatment regimen. For tissue acquisition during diagnostic biopsies, an extra biopsy of suspected malignant tissue was taken for this study during the procedure. For resection specimens, a small piece of tissue was sampled from the resected specimen at the tissue facility in the department of pathology. Tissue samples were immediately collected in +/+/+ organoid medium which consisted of advanced DMEM/F12 (AdDMEM/F12: Life Technologies, cat # 12634-034), supplemented with: 1× GlutaMAX (Thermofisher; Gibco, cat # 35050061), Penicillin–streptomycin (Life Technologies, cat # 15630-056), 10 mM HEPES (Life Technologies, cat # 15630-056) (+/+/+ medium) and 100 mg/mL Primocin (Invivogen, cat # ant-pm1). After transportation to the laboratory, organoid isolation was mostly performed on the same day as the tissue sampling, however in some cases isolation was performed within 3 days with an outlier of 10 days.

### Organoid Isolation

The PDO culturing in this study has been described previously [6, 15]. In short, tissue samples were mechanically cut into pieces (1–3 mm^2^) and digested for 20–40 min in 0.125% Trypsin (Sigma, cat # T1426) in +/+/+ medium supplemented with 10 μM Y-27632 (Abmole Bioscience, cat. no. M1817) at 37 °C. During incubation, mechanical force was used every 10 min to aid digestion by triturating the tissue pieces with a p1000 pipette. Tissue was subsequently triturated using a flame-sterilized pipette with a p10 tip on the end. Once pieces of tissue appeared macro- and microscopically dissociated, +/+/+ medium was topped up to 15 mL and the suspension was filtered through a 70 mM filter (Corning, cat # CLS431751-50EA). Tubes were centrifuged at 300 g, 5 min and the supernatant was aspirated. Using ice-cold 70% 10 mg/mL cold Cultrex growth factor reduced basement membrane extract (BME) type 2 (Trevigen, cat # 3533-010-02) in +/+/+ medium, the pellet was resuspended. BME/organoid suspension was plated in 10–20 mL droplets on the base of a preheated 48-well suspension culture plate (Greiner, cat # M9312). Plates were inverted and incubated at 37 °C for at least 15–30 min to allow solidification of BME. After solidification, pre-warmed culture medium supplemented with 10 μM Y-27632 and caspofungin (0.5 mg/mL, Sigma Aldrich) was added to the plates and they were incubated in a 37C/5% CO2 incubator. Two types of culture media were used for HNSCC organoids: a head neck (HN) cancer medium [6] or cervical squamous cell medium(M7) as previously described [22].

### Organoid Culturing

Organoids were subsequently grown from the primary material in culture media. All primary material was established on both HN and M7 medium to determine which medium was optimal for each organoid line. If an organoid line had an improved growth on a particular medium, this medium was subsequently used. HN and M7 medium were both supplemented with 0.5 mg/mL caspofungin for the first week of organoid culture and then removed. HN medium was also supplemented with 10 μM of Y-27632 for the first week of organoid culture and was then removed. However M7 medium was constantly supplemented with 10 μM of Y-27632. Medium was changed every 2–3 days and organoids were passaged between approximately 7 and 14 days after plating, depending on their growth rate.

To passage organoids, BME droplets were disrupted by resuspending the entire well content using a P1000 pipette. This was transferred to 15 mL Falcon tube, where up to 15 mL of +/+/+ was added and then centrifuged (300 g, 5 min). After centrifugation, the organoid pellet was resuspended in 1–3 mL TrypLE Express (Life Technologies, Carlsbad, CA, USA, cat. no. 12605-010) and incubated for 3–10 min at 37 °C. The digestion was constantly monitored by checking the tube under the microscope. Organoids were sheared mechanically using a P1000 pipette with an extra P10 tip placed on the tip. After organoids were disrupted into single cells, tubes were topped up to 15 mL of +/+/+ to inhibit the TrypLE digestion, and centrifuged. Supernatant was removed down to the pellet and cells were resuspended in 70% BME in +/+/+. The density of organoids were checked under the microscope before plating, if organoids were too dense, more 70% BME in +/+/+ was added. Multiple domes of 10–20 mL were plated on pre-heated suspension culture plates (Greiner, cat # M9312). Plates were inverted and incubated at 37C for at least 15 min for BME solidification. After solidification, pre-warmed HN or M7 media supplemented with 10 μM Y-27632 was added to the plates and they were incubated in a 37C/5% CO2 incubator. For cultures growing on HN media, Y-27632 was removed from the medium after 2–3 days and organoids were subsequently cultured in media without Y-27623. For M7 medium, Y-27632 was constantly in the media, and was therefore not removed after passaging.

To show that organoids contain tumor cells, the organoid cultures are exposed to nutlin-3a as described in Millen et al. [15] Nutlin-3a is an MDM2 antagonist that ceases growth of TP53 wildtype cells (non-tumor epithelial cells) but leaves TP53 mutant cells (tumor epithelial cells) unaffected. Cultures are exposed to nutlin-3a for a period of 7–10 days and it is determined to be a tumor-derived organoid if growth continues in the presence of nutlin-3a and normal-derived organoid if it dies in the presence of nutlin-3a.

### Clinical Data

Clinical data was extracted from the medical records. The following clinical parameters were collected: sex, age, prior cancer treatment status (defined as chemo- and/or radio-therapy). For tissue sampling details: type of sampling (biopsy or resection), date of sampling (i.e. time in days between sampling and organoid isolation), and for tumor details: tumor type, tumor location, TNM-stage [23] (if available pTNM otherwise cTNM), tumor diameter in centimeters, HPV-status, histopathological grade (Grade 1 well, Grade 2 moderately and Grade 3 poorly differentiated), presence of bone invasion, presence of angio-invasion, presence of perineural growth and growth pattern (cohesive vs. non-cohesive). HPV status was positive if it was pathologically confirmed. Bone invasion was positive if it was stated positive in the pathological report.

### Analysis

For the analysis, each organoid line was analyzed as a separate case. For 15 patients, more than one HNSCC tissue specimen was collected. For example: a biopsy was initially collected for diagnosis, a resection of the primary or recurrent tumor was subsequently performed. The primary outcome was organoid establishment (yes/no) and was defined as ‘successful' (or ‘yes’) if organoids reached Passage 1 (P1) and as ‘no’ if they did not grow and could not be passaged. P1 was defined as organoids that grew from isolation (P0) of primary tissue and were large enough to passage. Group differences were assessed for organoid establishment regarding the collected clinical variables on patient data, sampling data and tumor data.

The presence of epithelial cells in the organoid culture at P0 was available from the samples of 2019 onwards. After tissue processing, and upon plating the organoids (P0), the researcher recorded presence of epithelial cells with microscopic examination and presence was defined as ‘yes’ if either single cells or clumps of epithelial cells were present in the culture. If these were not observed, these were defined as “no epithelial cells present”.

### Statistics

The outcome variables are reported dichotomously. The continuous variables, age at surgery and tumor diameter, were split into two groups based on the median. The continuous variable ‘number of days between sampling and isolation’ was split into two groups (day 0 versus day 1 onwards). All of the other variables were nominal. Group differences per variable regarding organoid establishment and epithelial cell presence were assessed using the test of two proportions with a chi-square test of homogeneity. Bonferroni correction for multiple comparisons was executed. Statistical significance was considered if *p* < 0.0167 for patient factors (three comparisons), if *p* < 0.025 for sampling factors (two comparisons) and if *p* < 0.0083 for tumor factors (six comparisons).In case of an insufficient sample size, Fisher’s exact test was used. Statistical analysis was performed with SPSS Statistics (IBM Corp. Released 2020. IBM SPSS Statistics for Windows, Version 27.0. Armonk, NY: IBM Corp).

## Results

521 collected tissue samples for which organoid establishment was attempted were included in this study. Tissues that were not derived from squamous cell carcinoma were excluded from this analysis (*n* = 24) and organoid cultures that had a microscopically visible fungal or bacterial infection between isolation (P0) and P1 (*n* = 12). Tissues from 250 samples originated from primary tumor site (primary tumor *n* = 200, recurrent *n* = 17, second primary *n* = 26, third primary *n* = 6 and fifth primary *n* = 1), 27 originated from SCC-containing metastatic lymph nodes and 208 originated from normal mucosa adjacent to the HNSCC tumor. Patient characteristics are displayed in Table 1.

**Table 1:** Patient and tumor characteristics for corresponding organoid cultures

Characteristics		SCC tumor lines *N* = 250	Normal mucosa lines *N* = 208
Organoids	Established	133 (53.2)	141 (67.8)
Organoids	Established if epithelial cells present (*n* = 112)	86/112 (76.8)	
Tumor nature	Primary	200 (80.0)	
	2nd primary onwards	33 (13.2)	
	Recurrent	17 (6.8)	
Age (year)	Median (range)	68 (22–92)	69 (22–92)
Sex	Males	170 (68.0)	135 (64.9)
	Females	80 (32.0)	73 (35.1)
Pre treatment	No	208 (83.2)	168 (80.8)
	Yes ▪ Chemotherapy	1 (0.4)	2 (1.0)
	▪ Radiotherapy	27 (10.8)	27 (13.0)
	▪ Radiotherapy+Chemo	12 (4.8)	10 (4.8)
	▪ Radiotherapy+Cetuximab	2 (0.8)	1 (0.5)
	▪ Total	42 (16.8)	40 (19.2)
Sampling	Biopsy	75 (30.0)	12 (5.8)
	Resection	175 (70.0)	196 (94.2)
Organoid isolation day after surgery	Day 0	169 (75.8)	127 (70.9)
	Day 1	47 (21.1)	47 (26.3)
	Day 2–10	7 (3.1)	5 (2.8)
Tumor location	Oral cavity	151 (60.4)	
	Oropharynx	22 (8.8)	
	Hypopharynx	22 (8.8)	
	Larynx	48 (19.2)	
	Other^a^	7 (2.8)	
HPV status	Positive	12 (4.8)	
	Negative / not determined	238 (95.2)	
T-stage	T1	26 (10.4)	
	T2	88 (35.2)	
	T3	56 (22.4)	
	T4	80 (32.0)	
N-stage	N0	132 (52.8)	
	N1	28 (11.2)	
	N2	49 (19.6)	
	N3	41 (16.4)	
Bone invasion	No / Not reported	195 (78.0)	
	Yes	55 (22.0)	
Peri neural invasion	No	102 (53.1)	
	Yes	90 (46.9)	
Angio invasion	No	152 (85.4)	
	Yes	26 (14.6)	
Tumor differentia-tion	Well	11 (10.8)	
	Moderately	74 (72.5)	
	Poor	17 (16.7)	
Growth pattern	Cohesive	63 (36.4)	
	Non-Cohesive	110 (63.6)	
Tumor diameter	Median (range cm)	3.0 (0.7–9.50)	

Values are numbers with (%) unless otherwise stated

^a^Other comprises: Parotis (*n* = 1), Nasopharynx (*n* = 1), Nasal cavity (*n* = 5). Pathological TNM-status was used if possible otherwise the clinical TNM-stage was used

### HNSCC Organoid Establishment and Sample Purity

Organoids could be established in 133/250 (53.2%) primary tumor site tissues (Table 1). For the samples from 2019 onwards (*n* = 149) data about the presence of epithelial cells was confirmed. This was done on the day of isolation where epithelial cells were observed in the culture by bright-field microscopy in 112/149 (75.2%) of these samples. If there were epithelial cells present at P0, organoid establishment success rate increased to 76.8% (86/112 samples).

### HNSCC Organoid Establishment Correlation Factors

#### Patient Factors

**Fig. 1:**
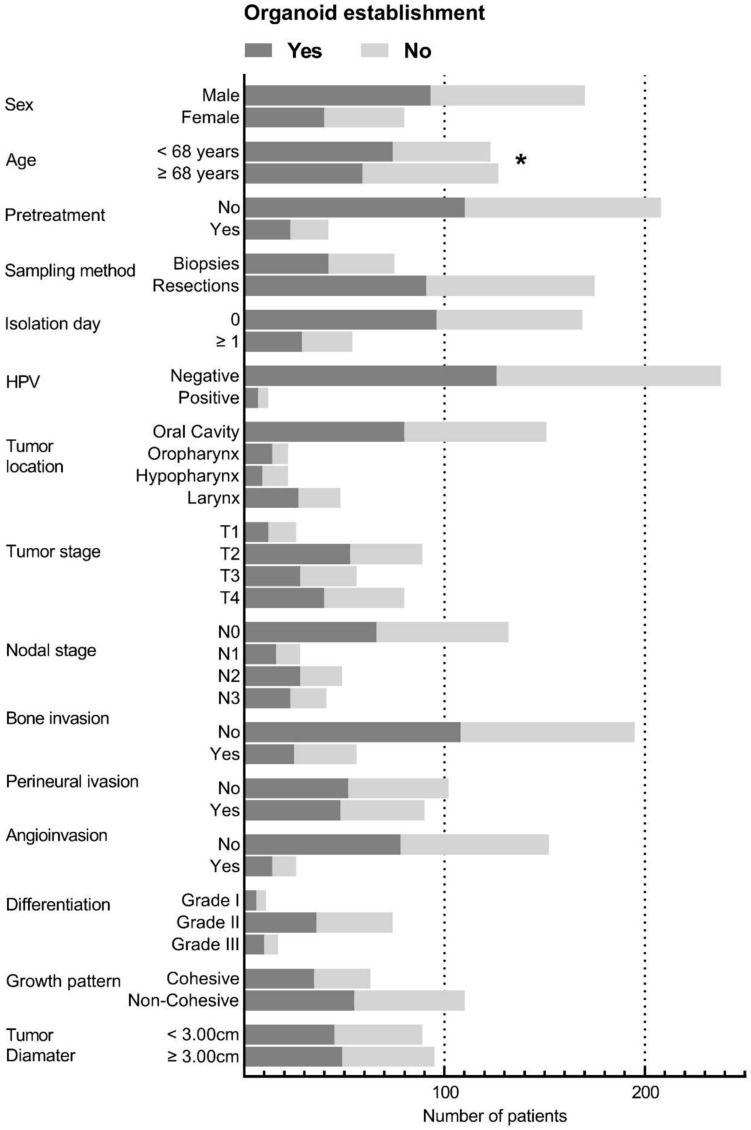
HNSCC group: organoid establishment correlating to clinical-, sampling- and tumor-parameters. X-axis shows number of patients. Y-axis shows different clinical parameters. *indicates a trend towards a statistical significant difference using the test of two proportions with a chi-square test of homogeneity. Pretreatment was defined as: patient received radiotherapy anywhere on the body and/or chemotherapy ever. Isolation day means: days between surgery and organoid isolation. Tumor and nodal stage according to the TNM criteria

PDO establishment tended to be more successful in patients who were younger than the median age of 68 years (74/123 (60.2%) vs. 59/127 (46.5%), *p* = 0.03) Table 2 and Fig. 1. There was no difference in successful establishment of organoids between males and females (93/170 (54.7%) vs. 40/80 (50.0%), *p* = 0.49) nor for patients that received previous anti-cancer treatment compared to patients without prior treatment (11/208 (52.9%) vs. 23/42 (54.8%), *p* = 0.82).

**Table 2:** HNSCCC group: analysis of organoid establishment per clinical factor

Factor with total number of cases available	Number of organoid lines established					Propor-tion Differ-ence	*p* value
	Groups (% of total within group)				Total (%)		
Patient factors							
Sex	Males	Females					
*n* = 250	93 (54.7)	40 (50.0)			133 (53.2)	0.047	0.49
Age (split median)	< 68 years	≥ 68 years					
*n* = 250	74 (60.2)	59 (46.5)			133 (53.2)	0.137	0.03
Pretreatment	No	Yes					
*n* = 250	110 (52.9)	23 (54.8)			133 (53.2)	0.019	0.82
Sampling factors							
Method	Biopsy	Resection					
*n* = 250	42 (56.0)	91 (52.0)			133 (53.2)	0.04	0.56
Days to org isolation	0	1–5					
*n* = 223	96 (56.8)	29 (53.7)			125 (56.0)	0.031	0.69
Tumor factors							
Tumor location^a^	Oral Cavity	Oropharynx	Hypopharynx	Larynx			
*n* = 243	80 (53.0)	14 (63.6)	9 (40.9)	27 (56.3)	130 (53.5)	NA	0.48
HPV status	Negative	Positive					
*n* = 250	126 (52.9)	7 (58.3)			133 (53.2)	0.054	0.72
T stage	T1	T2	T3	T4			
*n* = 250	12 (46.2)	53 (60.2)	28 (50.0)	40 (50.0)	133 (53.2)	NA	0.42
N stage	N0	N1	N2	N3			
*n* = 250	66 (50.0)	16 (57.1)	28 (57.1)	23 (56.1)	133 (53.2)	NA	0.76
Bone invasion	No	Yes					
*n* = 250	108 (55.4)	25 (45.5)			133 (53.2)	0.099	0.19
Peri neural invasion	No	Yes					
*n* = 192	52 (51.0)	48 (53.3)			100 (52.0)	0.023	0.74
Angio invasion	No	Yes					
*n* = 178	78 (51.3)	14 (53.8)			92 (51.7)	0.028	0.81
Differentiation	Grade I	Grade II	Grade III				
*n* = 102	6 (54.5)	36 (48.6)	10 (58.8)		52 (51.0)	NA	0.73
Growth pattern	Cohesive	Non-Cohesive					
*n* = 173	35 (55.6)	55 (50.0)			90 (52.0)	0.056	0.48
Tumor diameter	< 3 cm	≥ 3 cm					
*n* = 184	45 (50.6)	49 (51.6)			94 (51.0)	0.016	0.89

Tests of proportions used were the chi-square tests of homogeneity. Statistical significance after Bonferroni correction was considered if *p* < 0.0167 for patient factors (three comparisons), if *p* < 0.025 for sampling factors (two comparisons) and if *p* < 0.0083 for tumor factors (six comparisons) NA: Not applicable as there are several proportion differences between more than two groups

^a^Excluding location tumor ‘other’ (*n* = 7)

#### Tissue Sampling Factors

The majority of resection tumor specimens originated from the oral cavity, while the biopsy specimens were mostly derived from oropharyngeal, hypopharyngeal and laryngeal samples. There were no significant differences in PDO establishment between biopsy- and resection-specimens: 42/75 (56.0%) vs. 91/175 (52.0%), *p* = 0.56 nor between the organoids isolated at the day of surgery or later: 96/169 (56.8%) vs. 29/54 (53.7%), *p* = 0.69 (Table 2, Fig. 1).

#### Clinical Parameters

Of the 250 SCC samples, 151/250 (60.4%) tumor tissues originated from the oral cavity, 22/250 (8.8%) from the oropharynx, 22/250 (8.8%) from the hypopharynx, 48/250 (19.2%) from the larynx, and 7/250 (2.8%) from other tumor sites documented in Table 1. There were no differences regarding PDO establishment for: Tumor location, HPV-status, T-stage, N-stage, Bone invasion, perineural invasion, angioinvasion, tumor grade, growth pattern and tumor diameter (Table 2, Fig. 1).

#### Primary Tumor Site SCC vs Metastatic SCC

For SCC tissues from the primary tumor site, PDO establishment was slightly more successful compared to metastatic SCC, (133/250 (53.3%) vs. 12/27 (44.4%), *p* = 0.39). However, differences in proportions were not statistically significant.

#### Primary Tumor vs Secondary (or More) Primary and Recurrent

PDO establishment was not more successful for primary SCC tissues, compared to secondary (or more) or recurrent SCC, (108/200 (54%) vs. 25/50 (50%), *p* = 0.61).

### Normal Mucosa Organoid Establishment

Normal mucosa organoids could be established in 141/208 (67.8%) samples of tumor-adjacent epithelium. In this cohort, there were no differences in the success rate of PDO establishment for: sex, median age, pre-treated vs. untreated tumors and isolation on day of surgery or later (Table 3). There was a strong trend towards an improved success rate of PDOs from resection samples as compared to biopsies: 136/196 (69.4%) vs. 5/12 (41.7%), *p* = 0.06 (Table 3).

**Table 3:** Normal mucosa: analysis of organoid establishment per clinical factor

Factor with total number of cases available	Number of organoid lines established			Proportion Difference	p value
	Groups (% of total within group)		Total (%)		
Patient factors					
Sex	Males	Females			
*n* = 208	91 (67.4)	50 (68.5)	141 (67.8)	0.011	0.87
Age (split median)	< 69 years	≥ 69 years			
*n* = 208	73 (70.2)	68 (65.4)	141 (67.8)	0.048	0.46
Pretreatment	No	Yes			
*n* = 208	118 (70.2)	23 (57.5)	141 (67.8)	0.127	0.12
Sampling factors					
Method	Biopsy	Resection			
*n* = 208	5 (41.7)	136 (69.4)	141 (67.8)	0.277	0.06^a^
Days to org isolation^b^	0	1–5			
*n* = 179	86 (67.7)	39 (75.0)	125 (69.8)	0.073	0.35

Tests of proportions used were the chi-square tests of homogeneity. Statistical significance after Bonferroni correction was considered if *p* < 0.0167 for patient factors (three comparisons) and if *p* < 0.025 for sampling factors (two comparisons). NA: Not applicable as there are several proportion differences between more than two groups

^a^Fisher exact test because of low sample size biopsies

^b^Data of this parameters was not available for all tissues

### Tissue Sample Purity

**Fig. 2:**
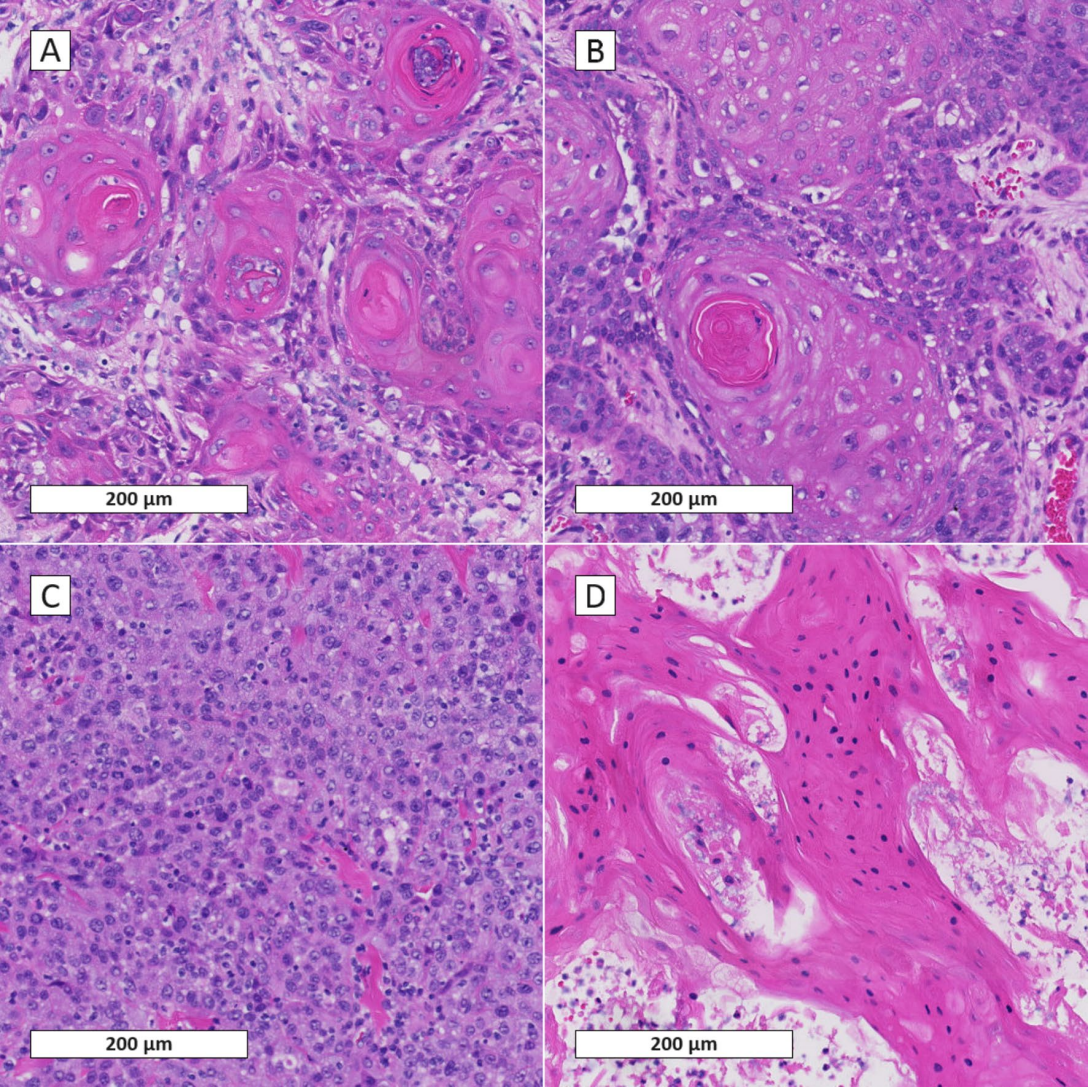
Hematoxylin and eosin stain of 4 tumor samples before organoid culturing; **A** tumor sample of Larynx; **B** tumor sample of oropharynx; **C** tumor sample of lymph node metastasis; **D** tumor sample of Oral Cavity; For **A** and **B** epithelial cells were present in the culture at P0; for **C** and **D** epithelial cells were not present in the culture at P0

In biopsies, there were significantly more epithelial cells present in the culture on the day of organoid isolation compared to resection specimens (45/52 (86.5%) vs. 67/97 (69.1%), *p* = 0.02, Table S1). Likewise, in the primary/local recurrent HNSCC samples, there were significantly more epithelial cells present in the culture on the day of isolation compared to the metastatic HNSCC samples (112/149 (75.2%) vs. 15/27 (55.6%), *p* = 0.04). There was a strong trend that cultures from patients below the median age of 68 years had more epithelial cells present in the culture on the day of isolation (82.6% vs 68.8%, *p* = 0.05, Table S1). The other clinical factors revealed no differences in presence of epithelial cells in the culture on the day of isolation, neither for SCC tissues nor for normal mucosa tissues (Table S1+ S2). Figure 2 displays H&E staining’s of four sampled tumor tissues before the start of organoid culturing. For Fig. 2A and B epithelial cells were present at P0, for Fig. 2C and D epithelial cells were not present in the culture at P0.

## Discussion

It is relevant to know if there are patient- and/or tumor-factors in tissue sampling that influence PDO establishment, as this could help future researchers navigate organoid biobanks. However, to date, there have been no studies in HNSCC to assess such factors. This study assessed clinical factors regarding PDO establishment and found a trend of PDO establishment being more successful in younger patients with HNSCC, below the median age of 68 years, although this was just not statistically significant (*p* = 0.03). This could be explained by the fact that cell division rates decrease with age [24]. The study of Larsen et al. published their supplemental data on age of 77 HNSCC organoids of which 12 were biobankable [25]. The mean age in deciles for the biobankable organoids was 6.67 and the mean age in deciles for the 65 non-biobankable organoids was 7.09. These findings are in line with our trend. In the whole cohort, which consisted of several organoid tumor types, age was not different for the biobankable and non-biobankable organoids [25]. For future HNSCC organoid studies, this could be considered, as this may support successful organoid outgrowth. This seems clinically applicable as elderly patients with comorbidities are already often refrained from chemotherapy [26]. As this cohort of patients only contained HNSCC, the median age associated with successful organoid outgrowth, may vary between other tumor types. Therefore, age as a factor for successful organoid culture should also be explored in other types of cancer.

There was no difference in PDO establishment between patients that received chemo—and/or radiotherapy compared to untreated patients. This is reassuring, as many cancer regimens include surgery as well as adjuvant systemic therapy, highlighting that the use of systemic therapy in HNSCC has no impact on organoid outgrowth. In line with this, Ooft et al. (colorectal cancer), Larsen et al. (several tumortypes) and Sharick et al. (pancreatic cancer) also did not find a correlation between previous chemotherapy and PDO outgrowth [18, 25, 27]. Interestingly, another study on colorectal cancer found a reduced PDO outgrowth if patients had received neoadjuvant chemoradiotherapy prior to tissue sampling compared to non-adjuvant and chemotherapy-only groups [28]. We did not find differences in PDO establishment between primary/locally recurrent tumors and metastatic tumors, which is in line with two other studies [17, 24]. Again, this is reassuring for future organoid studies that plan to establish organoids from metastatic tissue, as this may not affect outgrowth. Like Larsen et al. we did not find differences in PDO establishment based on tumor size [25].

We found a significantly higher presence of epithelial cells in biopsies compared to surgical resection specimens for HNSCC PDOs (86.5% vs 69.1%, *p* = 0.02), although there was no difference in PDO establishment itself. For prostate cancer, sampling with radical prostatectomy compared to transurethral resection of the prostate resulted in an improved PDO establishment for the radical prostatectomy group [29]. Likewise, for colorectal cancer, the rate of successful PDO culture was lower for endoscopic biopsies compared to surgical resection specimen [28]. Similar to our findings, differentiation grade did not influence PDO establishment in prostate cancer [29].

The influence of efficient sampling of the tumor on organoid establishment may be bigger than the influence of the clinical factors themselves. Here we show an increase in PDO establishment from 53.2% to 76.8% if organoid cultures were found to have epithelial cells present in the culture at P0. Moreover, in a subset analysis, we previously found an increase in the success rate of HNSCC organoids from 33.3% to 85.5% in cases where epithelial cells were present in the collected tissues, assessed by H&E staining of the tissue sample (*n* = 77, Fisher’s exact test, proportion 0.522, *p* < 0.001) [15]. For metastatic gastro-intestinal cancer organoids Vlachochiannis et al. also describe that the establishment rate strongly correlated with tumor cellularity in the original tissue biopsy [17]. Likewise in prostate organoids, a significant correlation between tumor cell percentage in the original tissue sample and prostate organoid establishment was reported [29]. This indicates that efficient sampling is important for optimizing PDO establishment. Figure 2 emphasizes the importance for efficient sampling.

Interestingly, in this analysis, we showed a statistically significant difference between biopsies and resections, with biopsy-derived organoid cultures typically having more epithelial cells present on the day of isolation compared to resections specimens. For biopsies, the sample is taken directly by the surgeon whereas for surgical resections, the tissue is removed from the body whereupon it is sampled at a later point by the pathology department. This difference in presence of epithelial cells on the day of isolation could be due to difficulty to recognize tumor versus normal tissue during sampling of the resected specimen vs. a tumor that is in situ in the body during a biopsy procedure. Additionally, we found epithelial cells more frequently in the culture at P0 for primary tumor tissues compared to metastatic tumors in lymph nodes (75.5% vs 55.6%, *p* = 0.03). A tendency that the success rate of PDO establishment is better for primary tumor site samples, suggests sampling efficiency is better at the primary tumor site compared to HNSCC lymph node metastasis.

In this study, organoids were deemed successful if they reached P1, and most organoids included in the analysis were started on both HN and M7 media types to determine the optimal media for each organoid line. Therefore, the effect of the media composition on successful outgrowth is not a factor in our analysis, and it is more important if a culture has epithelial cells present on day of isolation or not. Larsen et al. have investigated the effect that various growth factors have on functional growth and phenotypes in tumor organoids derived from various tumor types. They found that although EGF stimulated proliferation in most cultures, there was no significant difference in growth between the five various media conditions that were tested. In particular, they assessed the success rate of head and neck organoids when established on complete versus minimum media type, and found no difference.

In conclusion, this study found a positive trend between age and successful organoid outgrowth in patients with HNSCC younger than 68 years and emphasizes the value of efficient tumor sampling to achieve successful PDO establishment. This study highlights the importance of future organoid studies to evaluate clinical factors that may influence organoid outgrowth, and to investigate this in other tumor types.

## Supplementary Information

Below is the link to the electronic supplementary material.

Supplementary file1 (DOCX 19 KB)

## Data Availability

The datasets generated during and/or analysed during the current study are available from the corresponding author on reasonable request.

## References

[1] Johnson DE, Burtness B, Leemans CR, Lui VWY, Bauman JE, Grandis JR (2020) Head and neck squamous cell carcinoma. Nat Rev Dis Prim 6(1):92. 10.1038/s41572-020-00224-3 33243986 10.1038/s41572-020-00224-3 PMC7944998

[2] Gavrielatou N, Doumas S, Economopoulou P, Foukas PG, Psyrri A (2020) Biomarkers for immunotherapy response in head and neck cancer. Cancer Treat Rev 84:101977. 10.1016/j.ctrv.2020.101977 32018128 10.1016/j.ctrv.2020.101977

[3] Hsieh JCH, Wang HM, Wu MH et al (2019) Review of emerging biomarkers in head and neck squamous cell carcinoma in the era of immunotherapy and targeted therapy. Head Neck 41(S1):19–45. 10.1002/hed.25932 31573749 10.1002/hed.25932

[4] de Kort WWB, Spelier S, Devriese LA, van Es RJJ, Willems SM (2021) Predictive value of EGFR-PI3K-AKT-mTOR-pathway inhibitor biomarkers for head and neck squamous cell carcinoma: a systematic review. Mol Diagnosis Ther 25(2):123–136. 10.1007/s40291-021-00518-6 10.1007/s40291-021-00518-6 PMC7956931 33686517

[5] Drost J, Clevers H (2018) Organoids in cancer research. Nat Rev Cancer 18(7):407–418. 10.1038/s41568-018-0007-6 29692415 10.1038/s41568-018-0007-6

[6] Driehuis E, Kolders S, Spelier S et al (2019) Oral mucosal organoids as a potential platform for personalized cancer therapy. Cancer Discov 9(7):852–871. 10.1158/2159-8290.CD-18-1522 31053628 10.1158/2159-8290.CD-18-1522

[7] Tuveson D, Clevers H (2019) Cancer modeling meets human organoid technology. Science 364(6444):952–955. 10.1126/science.aaw6985 31171691 10.1126/science.aaw6985

[8] Sato T, Vries RG, Snippert HJ et al (2009) Single Lgr5 stem cells build crypt-villus structures in vitro without a mesenchymal niche. Nature 459(7244):262–265. 10.1038/nature07935 19329995 10.1038/nature07935

[9] Kim M, Mun H, Sung CO et al (2019) Patient-derived lung cancer organoids as in vitro cancer models for therapeutic screening. Nat Commun 10(1):3991. 10.1038/s41467-019-11867-6 31488816 10.1038/s41467-019-11867-6 PMC6728380

[10] Yan HHN, Siu HC, Law S et al (2018) A comprehensive human gastric cancer organoid biobank captures tumor subtype heterogeneity and enables therapeutic screening. Cell Stem Cell 23(6):882-897.e11. 10.1016/j.stem.2018.09.016 30344100 10.1016/j.stem.2018.09.016

[11] Van De Wetering M, Francies HE, Francis JM et al (2015) Prospective derivation of a living organoid biobank of colorectal cancer patients. Cell 161(4):933–945. 10.1016/j.cell.2015.03.053 25957691 10.1016/j.cell.2015.03.053 PMC6428276

[12] Sachs N, de Ligt J, Kopper O et al (2018) A living Biobank of breast cancer organoids captures disease heterogeneity. Cell 172(1–2):373-386.e10. 10.1016/j.cell.2017.11.010 29224780 10.1016/j.cell.2017.11.010

[13] Hill SJ, Decker B, Roberts EA et al (2018) Prediction of DNA repair inhibitor response in short-term patient-derived ovarian cancer organoids. Cancer Discov 8(11):1404–1421. 10.1158/2159-8290.CD-18-0474 30213835 10.1158/2159-8290.CD-18-0474 PMC6365285

[14] Hou S, Tiriac H, Sridharan BP et al (2018) Advanced development of primary pancreatic organoid tumor models for high-throughput phenotypic drug screening. SLAS Discov 23(6):574–584. 10.1177/2472555218766842 29673279 10.1177/2472555218766842 PMC6013403

[15] Millen R, De Kort WWB, Koomen M et al (2023) Patient-derived head and neck cancer organoids allow treatment stratification and serve as a tool for biomarker validation and identification. Med 4(5):290-310.e12. 10.1016/j.medj.2023.04.003 37178682 10.1016/j.medj.2023.04.003

[16] Wensink GE, Elias SG, Mullenders J et al (2021) Patient-derived organoids as a predictive biomarker for treatment response in cancer patients. npj Precis Oncol 5(1):30. 10.1038/s41698-021-00168-1 33846504 10.1038/s41698-021-00168-1 PMC8042051

[17] Vlachogiannis G, Hedayat S, Vatsiou A et al (2018) Patient-derived organoids model treatment response of metastatic gastrointestinal cancers. Science 359(6378):920–926. 10.1126/science.aao2774 29472484 10.1126/science.aao2774 PMC6112415

[18] Ooft SN, Weeber F, Dijkstra KK et al (2019) Patient-derived organoids can predict response to chemotherapy in metastatic colorectal cancer patients. Sci Transl Med 11(513):eaay2574. 10.1126/scitranslmed.aay2574 31597751 10.1126/scitranslmed.aay2574

[19] Yao Y, Xu X, Yang L et al (2020) Patient-derived organoids predict chemoradiation responses of locally advanced rectal cancer. Cell Stem Cell 26(1):17-26.e6. 10.1016/j.stem.2019.10.010 31761724 10.1016/j.stem.2019.10.010

[20] Kondo J, Inoue M (2019) Application of cancer organoid model for drug screening and personalized therapy. Cells 8(5):470. 10.3390/cells8050470 31108870 10.3390/cells8050470 PMC6562517

[21] Foo MA, You M, Chan SL et al (2022) Clinical translation of patient-derived tumour organoids- bottlenecks and strategies. Biomark Res 10(1):10. 10.1186/s40364-022-00356-6 35272694 10.1186/s40364-022-00356-6 PMC8908618

[22] Lõhmussaar K, Oka R, Espejo Valle-Inclan J et al (2021) Patient-derived organoids model cervical tissue dynamics and viral oncogenesis in cervical cancer. Cell Stem Cell 28(8):1380-1396.e6. 10.1016/j.stem.2021.03.012 33852917 10.1016/j.stem.2021.03.012

[23] Huang SH, O’Sullivan B (2017) Overview of the 8th edition TNM classification for head and neck cancer. Curr Treat Options Oncol 18(7):40. 10.1007/s11864-017-0484-y 28555375 10.1007/s11864-017-0484-y

[24] Tomasetti C, Poling J, Roberts NJ et al (2019) Cell division rates decrease with age, providing a potential explanation for the age-dependent deceleration in cancer incidence. Proc Natl Acad Sci USA 116(41):20482–20488. 10.1073/pnas.1905722116 31548407 10.1073/pnas.1905722116 PMC6789572

[25] Larsen BM, Kannan M, Langer LF et al (2021) A pan-cancer organoid platform for precision medicine. Cell Rep 36(4):109429. 10.1016/j.celrep.2021.109429 34320344 10.1016/j.celrep.2021.109429

[26] Bahig H, Fortin B, Alizadeh M et al (2015) Predictive factors of survival and treatment tolerance in older patients treated with chemotherapy and radiotherapy for locally advanced head and neck cancer. Oral Oncol 51(5):521–528. 10.1016/j.oraloncology.2015.02.097 25797461 10.1016/j.oraloncology.2015.02.097

[27] Sharick JT, Walsh CM, Sprackling CM et al (2020) Metabolic heterogeneity in patient tumor-derived organoids by primary site and drug treatment. Front Oncol 10:553. 10.3389/fonc.2020.00553 32500020 10.3389/fonc.2020.00553 PMC7242740

[28] Zeng YL, Wang SD, Li YR et al (2023) Analysis of factors influencing the success rate of organoid culture in 1231 cases of colorectal cancer. Zhonghua Wei Chang Wai Ke Za Zhi 26(8):780–786. 10.3760/cma.j.cn441530-20221128-00499 37574295 10.3760/cma.j.cn441530-20221128-00499

[29] Servant R, Garioni M, Vlajnic T et al (2021) Prostate cancer patient-derived organoids: detailed outcome from a prospective cohort of 81 clinical specimens. J Pathol 254(5):543–555. 10.1002/path.5698 33934365 10.1002/path.5698 PMC8361965

