# Supplementary material for: Clinicopathological Factors as Predictors for Establishment of Patient Derived Head and Neck Squamous Cell Carcinoma Organoids
Source: Head Neck Pathol. 2024 Jun 28;18(1):59. doi: 10.1007/s12105-024-01658-x (PMC11213837; doi:10.1007/s12105-024-01658-x)
Supplement: Supplementary file 1 — Supplementary file1 (DOCX 19 KB) [file 12105_2024_1658_MOESM1_ESM.docx]

**Table S1 HNSCCC group:** Analysis of epithelial cell presence on day of isolation per clinical factor. Tests of proportions used were the chi-square tests of homogeneity. Statistical significance after Bonferroni correction was considered if p<0.0167 for patient factors (three comparisons), if p<0.025 for sampling factors (two comparisons) and if p<0.0083 for tumor factors (six comparisons) NA: Not applicable as there are several proportion differences between more than two groups.

*fisher exact test because of low sample size.

| Factor with total number of cases available | Number of cultures with epithelial cell presence | | | | | | | | Propor-tion  Differ-ence | p value |
| --- | --- | --- | --- | --- | --- | --- | --- | --- | --- | --- |
|  | Groups (% of total within group) | | | | | | Total (%) | |  |  |
| **Patient Factors** |  |  | |  | |  | |  |  |  |
| Sex | Males | Females |  | |  | |  | |  |  |
| n=149 | 78 (74.3) | 34 (77.3) |  | |  | | 112 (75.2) | | 0.03 | 0.70 |
| Age | < 68 years | ≥ 68 years |  | |  | |  | |  |  |
| n=149 | 57 (82.6) | 55 (68.8) |  | |  | | 112 (75.2) | | 0.138 | 0.05 |
| Pretreatment | No | Yes |  | |  | |  | |  |  |
| n=149 | 87 (74.4) | 25 (78.1) |  | |  | | 112 (75.2) | | 0.037 | 0.66 |
| **Sampling factors** |  |  |  | |  | |  | |  |  |
| Method | Biopsy | Resection |  | |  | |  | |  |  |
| n=149 | 45 (86.5) | 67 (69.1) |  | |  | | 112 (75.2) | | **0.174** | **0.02** |
| Days to org isolation | Day 0 | Day 1-5 |  | |  | |  | |  |  |
| n=146 | 88 (77.9) | 23 (69.7) |  | |  | | 111 (76.0) | | 0.082 | 0.33 |
| **Tumor factors** |  |  |  | |  | |  | |  |  |
| Tumor location | Oral Cavity | Oropharynx | Hypopharynx | | Larynx | |  | |  |  |
| n=142 | 53 (69.7) | 12 (85.7) | 15 (88.2) | | 28 (80.0) | | 108 (76.0) | | NA | 0.25 |
| HPV status | Negative | Positive |  | |  | |  | |  |  |
| n=149 | 107 (74.8) | 5 (83.3) |  | |  | | 112 (75.2) | | 0.085 | 1.00* |
| T stage | T1 | T2 | T3 | | T4 | |  | |  |  |
| n=149 | 12 (92.3) | 37 (72.5) | 26 (74.3) | | 37 (74.0) | | 112 (75.2) | | NA | 0.56* |
| N stage | N0 | N1 | N2 | | N3 | |  | |  |  |
| n=149 | 58 (74.4) | 15 (78.9) | 14 (70.0) | | 25 (78.1) | | 112 (75.2) | | NA | 0.89* |
| Bone invasion | No | Yes |  | |  | |  | |  |  |
| n=149 | 86 (78.2) | 26 (66.7) |  | |  | | 112 (75.2) | | 0.115 | 0.15 |
| Peri neural invasion | No | Yes |  | |  | |  | |  |  |
| n=110 | 37 (64.9) | 40 (75.5) |  | |  | | 77 (70.0) | | 0.106 | 0.23 |
| Angio invasion | No | Yes |  | |  | |  | |  |  |
| n=101 | 56 (69.1) | 13 (65.0) |  | |  | | 69 (68.3) | | 0.041 | 0.72 |
| Differentiation | Grade 1 | Grade 2 | Grade 3 | |  | |  | |  |  |
| n=79 | 3 (42.9) | 41 (70.7) | 10 (71.4) | |  | | 54 (68.4) | | NA | 0.36* |
| Growth pattern | Cohesive | Non-Cohesive | | | | |  | |  |  |
| n=96 | 18 (66.7) | 49 (71.0) |  | |  | | 67 (69.8) | | 0.043 | 0.68 |
| Tumor diameter | < 3.15cm | ≥ 3.15 cm |  | |  | |  | |  |  |
| n=104 | 34 (65.4) | 37 (71.2) |  | |  | | 71 (68.3) | | 0.058 | 0.53 |

**Table S2 Normal Mucosa:** Analysis of epithelial cell presence on day of isolation per clinical factor. Tests of proportions used were the chi-square tests of homogeneity. Statistical significance after Bonferroni correction was considered if p<0.0167 for patient factors (three comparisons) and if p<0.025 for sampling factors (two comparisons). NA: Not applicable as there are several proportion differences between more than two groups.

| Factor with total number of cases available | Number of cultures with epithelial cell presence | | | | Proportion  Difference | P value |
| --- | --- | --- | --- | --- | --- | --- |
|  | Groups  (% of total within group) | | Total (%) | |  |  |
| **Patient Factors** |  | |  |  |  |  |
| Sex | Males | Females |  | |  |  |
| n=133 | 65 (71.4) | 34 (81.0) | 99 (74.4) | | 0.096 | 0.24 |
| Age (split median) | < 69 years | ≥ 69 years |  | |  |  |
| n=133 | 47 (74.6) | 52 (74.3) | 99 (74.4) | | 0.003 | 0.97 |
| Pretreatment | No | Yes |  | |  |  |
| n=133 | 78 (76.5) | 21 (67.7) | 99 (74.4) | | 0.088 | 0.33 |
| **Sampling factors** |  |  |  | |  |  |
| Method | Biopsy | Resection |  | |  |  |
| n=133 | 7 (63.6) | 92 (75.4) | 99 (74.4) | | 0.118 | 0.47* |
| Days to org isolation | 0 | 1-5 |  | |  |  |
| n=130 | 64 (69.6) | 32 (84.2) | 96 (73.8) | | 0.146 | 0.08 |

*fisher exact test because of low sample size.
